# Ionospheric Response to the Severe Geomagnetic Storm of 19–21 January 2026 over Almaty (Kazakhstan) According to the Digisonde and GPS Data

**DOI:** 10.3390/s26185935

**Published:** 2026-09-19

**Authors:** Galina Gordiyenko, Yurii Litvinov, Murat Zhiganbayev, Valentina Grichshenko, Serik Nurakynov, Alexey Andreyev, Vlad Zhigalov

**Affiliations:** 1 Institute of the Ionosphere, Almaty 050020, Kazakhstan; ggordiyenko@mail.ru (G.G.); litvinov@ionos.kz (Y.L.); labreab@mail.ru (V.G.); nurakynov@gmail.com (S.N.); alexey.andreev@ionos.kz (A.A.); zhigalov@gmail.com (V.Z.); 2 JSC “Academy of Civil Aviation”, Almaty 050039, Kazakhstan; 3 Department of Electronics, Telecommunications and Space Technologies, Institute of Automation and Information Technologies, Satbayev University, Almaty 050013, Kazakhstan

**Keywords:** ionospheric monitoring, Digisonde DPS-4D, GPS-TEC, space weather, geomagnetic storm, mid-latitude ionosphere, foF2 dynamics

## Abstract

**Highlights:**

**What are the main findings?**

- We present initial operational results from observing the ionosphere during the severe geomagnetic storm of 19–21 January 2026, using the newly installed Digisonde Portable Sounder (DPS-4D) at the Almaty station (43.25° N, 76.92° E, Central Asia, Kazakhstan).
- A synergistic combination of high-resolution ionosonde measurements and GPS-based Total Electron Content (TEC) analysis enabled a detailed investigation of ionospheric structural dynamics during the storm.

**What are the implications of the main findings?**

- The deployment of the DPS-4D sounder expands global ionospheric monitoring capabilities within the Global Ionospheric Radio Observatory (GIRO) network.
- The observations provide valuable empirical evidence for characterizing the temporal development of regional ionospheric storms and their responses to geomagnetic activity.

**Abstract:**

We present observations of the ionospheric response to the severe geomagnetic storm of 19–21 January 2026, obtained using a Digisonde DPS-4D and GPS-based vertical Total Electron Content (TEC) measurements over Almaty, Kazakhstan. Solar wind parameters, geomagnetic indices (AE and SYM-H), and Interplanetary Magnetic Field (IMF) components were analyzed to characterize the storm drivers. The ionospheric response to the geomagnetic storm of 12–13 November 2025 was additionally analyzed to provide an overall picture of the ionospheric effects within the specified latitude–longitude region. The evolution of the ionospheric disturbances revealed highly complex processes. The critical frequency and total electron content during the severe geomagnetic storms exhibited morphological similarities, reflecting a general response to geomagnetic storms, as well as notable differences in their storm-time responses and in the observed percentage deviations of foF2 and TEC relative to reference-day baselines. A combined observational approach provides a more complete picture of the ionospheric response during extreme space weather events.

## 1. Introduction

Geomagnetic storms represent a major driver of space weather, exerting profound influences on the coupled magnetosphere-ionosphere-thermosphere system [1,2,3,4,5]. These events induce large-scale variations in ionospheric plasma density, thermospheric neutral composition, and global electrodynamics, leading to complex spatial and temporal perturbations. Studying storm-time ionospheric behavior remains essential for advancing fundamental upper-atmospheric physics and mitigating space weather impacts on modern technological infrastructure, such as Global Navigation Satellite Systems (GNSS), high-frequency (HF) communications, and power grids [6,7,8,9,10]. Severe storms cause enhanced satellite drag, ionospheric scintillation, and thermospheric heating, as routinely monitored by instruments like the Global-Scale Observations of the Limb and Disk (GOLD) mission [11].

Recently, the authors [4,12] undertook a large-scale statistical analysis of the global ionospheric response to geomagnetic storms, using data from the global network of navigation receivers. They identified a pronounced general dependence of the ionospheric response on latitude and longitude, with an emphasis on the latitude dependence of the main phase onset [4] and the regional total electron content [12]. Meanwhile, Chernigovskaya et al. [13] investigated the dynamics and mechanisms of ionospheric disturbances during geomagnetic storms, using data from various instruments, including ionosondes, GPS, and radar. The authors analyzed how the ionosphere changes at different latitudes, linking these changes to specific mechanisms, such as thermospheric winds, electric fields, and photoionization. They demonstrated that ionospheric storm development depends on a number of factors, including the driver type (CME or HSS), background conditions, and pre-storm anomalies. Evans et al. [11] were the first to thoroughly examine how the extreme geomagnetic storm of 11–12 May 2024 affected the thermosphere and linked the dynamics of the ionospheric storm to changes in the composition and temperature of the thermosphere. An unusual space weather scenario in February 2022 (“weak geomagnetic storm—loss of 38 Starlink satellites”) is reported in the paper by Kataoka et al. [14]. The cause was found to be an atypical structure of the coronal mass ejection (CME), which was not accounted for in the standard environmental forecast model and ultimately led to a fatal outcome. A multiparametric analysis of LAERT ionosonde data (installed on the Ionosphere-M satellites) during the storm of 12–14 November 2025 is presented in [15]. The authors demonstrated how complex and ambiguous the ionospheric response to large geomagnetic storms is and that analysis of such events helps to better understand the physical mechanisms and, ultimately, improve space weather models.

Thus, the authors of the paper [4,11,12,13,14,15] demonstrated that ionospheric responses to geomagnetic storms are not universal; their sign and amplitude depend strongly on the latitude, longitude, and local time of the geomagnetic storm’s onset. The importance of studying the regional characteristics of ionospheric disturbances and background conditions (pre-storm anomalies, driver type, structure, etc.) under which an ionospheric storm develops, when even a moderate geomagnetic storm can produce a strong ionospheric effect, is demonstrated.

The location of the Almaty station—the only station in Central Asia—provides access to critical ionospheric observation data for the region, which are essential for studying large-scale space weather processes.

Section 2 outlines the instrumentation, data sources, and methodology. Section 3 details the observational results. Section 4 presents the discussion and concluding remarks.

## 2. Materials and Methods

The ionospheric response to the geomagnetic storm of 19–21 January 2026, over the Almaty station (43.25° N, 76.92° E), was analyzed using data from the Lowell Digital Portable Sounder 4D (DPS-4D; ref. [16]), which was installed at the Almaty station in November 2025. Complementary vertical Total Electron Content (TEC) data were retieved from the nearby CHUM GPS receiver (Chumish, 43.90° N, 74.75° E). The primary analysis period spans 19–23 January 2026, covering the storm and early recovery phases. 18 January 2026, characterized by relatively low geomagnetic activity (Kp = 3.0, Ap = 15), was selected as a reference day representing relatively quiet geomagnetic conditions.

The Digisonde DPS-4D, developed by Lowell Digisonde International (LDI) and the UMass Lowell Center for Atmospheric Research (UMassLCAR), executes automated digital signal generation, echo reception, and real-time trace inversion, https://digisonde.com/ (accessed on 14 September 2026). The system determines standard ionospheric parameters (critical frequency of the F2-layer (foF2), minimum virtual height of the ordinary wave F trace (h′F), and peak height of the F2-layer (hmF2)), calculates electron density profiles N(h), and measures reflection characteristics across 0.5–30 MHz (group delay, amplitude, phase, polarization, Doppler spectrum, and arrival angles). By resolving echo arrival angles, the DPS-4D constructs directional “sky maps” and derives horizontal plasma drift vectors. High-rate ionogram data are automatically transmitted to the Global Ionospheric Radio Observatory (GIRO) network also developed by Lowell Digisonde International (LDI) and the UMass Lowell Center for Atmospheric Research (UMassLCAR) https://giro.uml.edu/ionoweb (accessed on 14 September 2026).

TEC estimates are derived using the IONOLAB-TEC framework, employing the Reg-Est algorithm for dual-frequency GPS receiver data with a 2.5 min temporal resolution. The estimates are obtained from phase-delay information that inherently includes automatic baseline and ambiguity resolution algorithms; IONOLAB-TEC uses IONOLAB-BIAS for receiver differential code bias estimation that resolves the single station differential code bias problem with high accuracy and computational efficiency. The IONOLAB-TEC code can be used from www.ionolab.org, (accessed on 14 September 2026) (Department of Electrical and Electronics Engineering, Hacettepe University, Ankara, Türkiye, accessed on 14 September 2026), or it can be downloaded to individual computers [17,18,19,20].

Interplanetary parameters—including total IMF intensity (Bt), IMF Bz component, solar wind speed (Vsw), and dynamic pressure (Psw)—along with geomagnetic indices (AE and SYM-H) are retrieved at 1 min resolution from the NASA/GSFC OMNIWeb database (https://omniweb.gsfc.nasa.gov/form/omni_min.html, accessed on 14 September 2026). Space weather contextual data were obtained from the NOAA Space Weather Prediction Center (SWPC) and SpaceWeather.com archives as ftp://ftp.ngdc.noaa.gov/STP/swpc_products/weekly_reports/ (accessed on 14 September 2026) and http://spaceweather.com (accessed on 14 September 2026).

The purpose of this study is to investigate the ionospheric response to the severe geomagnetic storm of 19–21 January 2026 (SYM-Hmin = −252 nT) by integrating DPS-4D digisonde soundings with GPS-derived TEC observations within the same latitude–longitude sector. For this purpose, all ionogram data, including the autoscaled critical frequency foF2, the minimum virtual height of the F layer, and the peak height hmF2, obtained at the standard 5 min cadence, were analyzed. The consistency of the automatically derived data with the results of direct ionogram analysis is assessed. When erroneous or ambiguous values are identified, an additional analysis of the temporal sequence of adjacent ionograms is performed. This procedure makes it possible to identify cases in which the results of automatic scaling require adjustment. Where necessary, the automatically determined parameters are visually inspected and manually corrected. For ionograms exhibiting diffuse echoes (e.g., spread-F), the results of automated processing are used only when they are consistent with the established estimates. If the automated determination is considered erroneous or ambiguous, the corresponding parameter is scaled manually in accordance with the standard rules for ionogram interpretation specified in the URSI handbook [21]. In the case of a blanketing sporadic-E layer (Es), if a reliable trace of the overlying reflection cannot be identified, the corresponding parameter for the given ionogram is not estimated and is classified as “data missing.” The possibility of recovering missing values is considered only at a subsequent stage of time-series analysis; where sufficient justification exists, interpolation is performed based on the temporal behavior of the parameter before and after the data gap. Thus, interpolation is not applied during the primary processing of individual ionograms, thereby avoiding the introduction of artificially generated values into the original dataset.

## 3. Observations

### 3.1. Geomagnetic Storm of 19–21 January 2026

On 18 January 2026 (18:00 UT), active region AR4341 produced a long-duration X1.9 solar flare associated with a full-halo Coronal Mass Ejection (CME). The CME impact at Earth on 19 January initiated a severe G4 geomagnetic storm (SYM-Hmin = −252 nT) spanning 19–20 January, followed by a G3 storm stage on 21 January. Secondary activity (G1 level) persisted through 22–23 January due to CME trailing structures and the arrival of a positive-polarity Coronal Hole High-Speed Stream (CH HSS).

**Figure 1:**
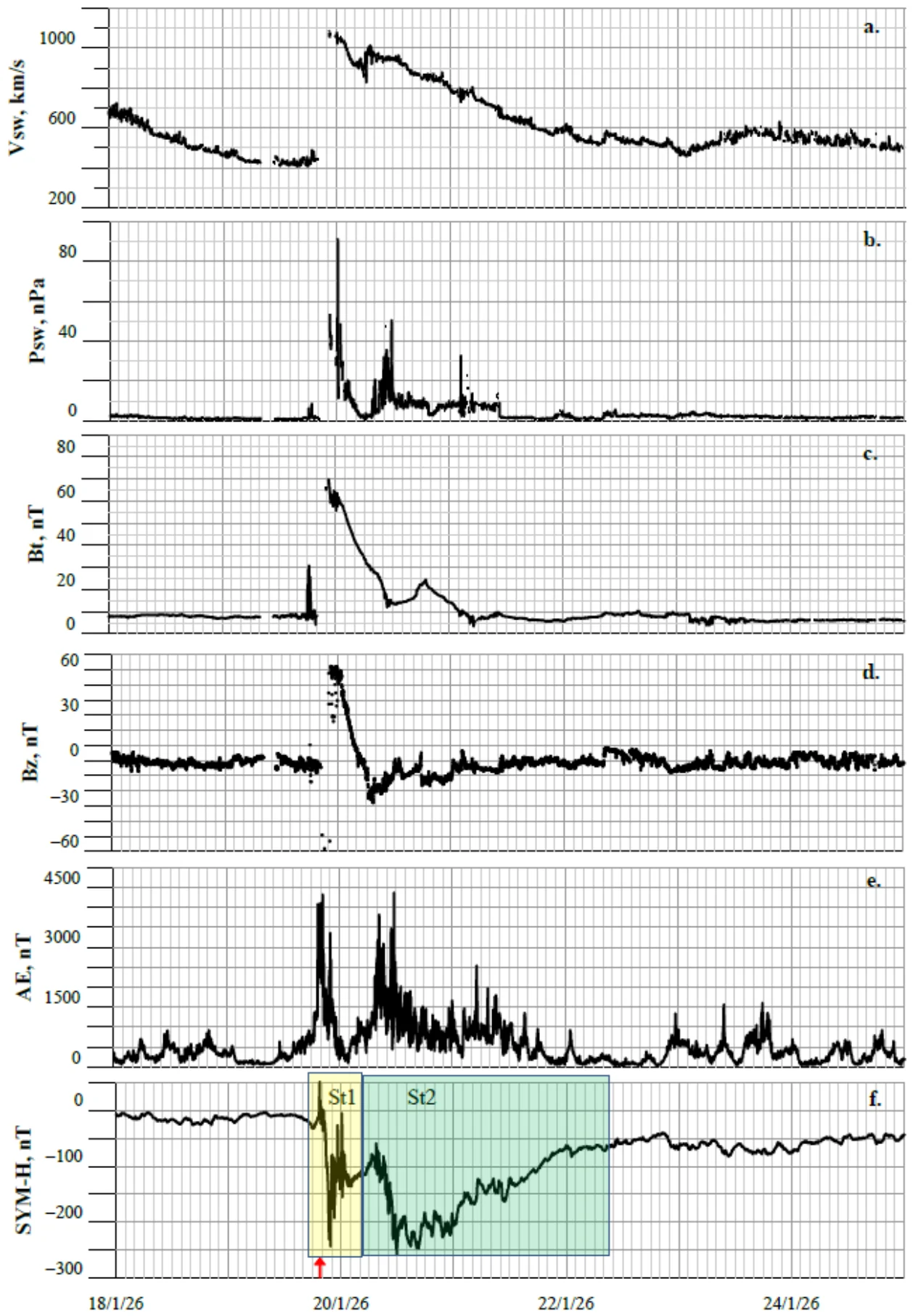
(**a**) Time series (UT) of solar wind speed Vsw, (**b**) dynamic pressure Psw, (**c**) IMF components Bt and (**d**) Bz, (**e**) auroral electrojet index AE, and (**f**) SYM-H index for 18–24 January 2026. Symbol ↑ indicates the onset of the storm; St1 and St2 are stages of the storm development (highlighted in yellow and green).

Figure 1 illustrates the helio-geophysical drivers from 18 to 24 January 2026. The storm’s sudden commencement (SSC) occurred at 19:24 UT on 19 January, marked by a sharp impulse (ΔSYM-H = +51 nT). Data gaps are present between 19:54 UT and 22:24 UT on 19 January in the OMNI solar wind stream records due to sensor saturation. However, available measurements indicate that Vsw surged to ∼1100 km/s, accompanied by high dynamic pressure (Psw) and elevated Bt. During the initial storm phase, IMF Bz exhibited rapid, large-amplitude oscillations between −60 nT and +60 nT. High AE values indicated sustained high-latitude magnetospheric energy input. The storm exhibited a complex, two-stage main phase development (St1 and St2):

*Stage St1:* Main phase MP1 commenced at 19:24 UT on 19 January, reaching a local SYM-H minimum of −243 nT at 21:42 UT (duration ≈ 2 h 18 min), driven by prolonged negative Bz. A rapid partial recovery followed, modulated by SYM-H oscillations that tracked fluctuations in the AE index.

*Stage St2:* Re-intensification occurred at 09:23 UT on 20 January during the recovery phase of St1, driving SYM-H to its absolute minimum of −252 nT. This secondary drop was associated with embedded CME magnetic structures that maintained a sustained southward IMF Bz (<−10 nT, peaking at −30 nT) until ∼10:00 UT on 21 January. In this event, the minimum SYM-H occurred approximately 4 h after the peak negative Bz.

For the event considered here, after SYM-H reached a stable level of −50 nT, the subsequent evolution of the storm was accompanied by continued AE activity. The temporal evolution of SYM-H and AE showed a qualitative correspondence, with the minimum SYM-H occurring approximately 3 h after the maximum AE value. These temporal relationships are presented here as descriptive features of the analyzed event and should not be interpreted as statistically established correlations or general physical dependencies.

### 3.2. Ionospheric Responses over Almaty

**Figure 2:**
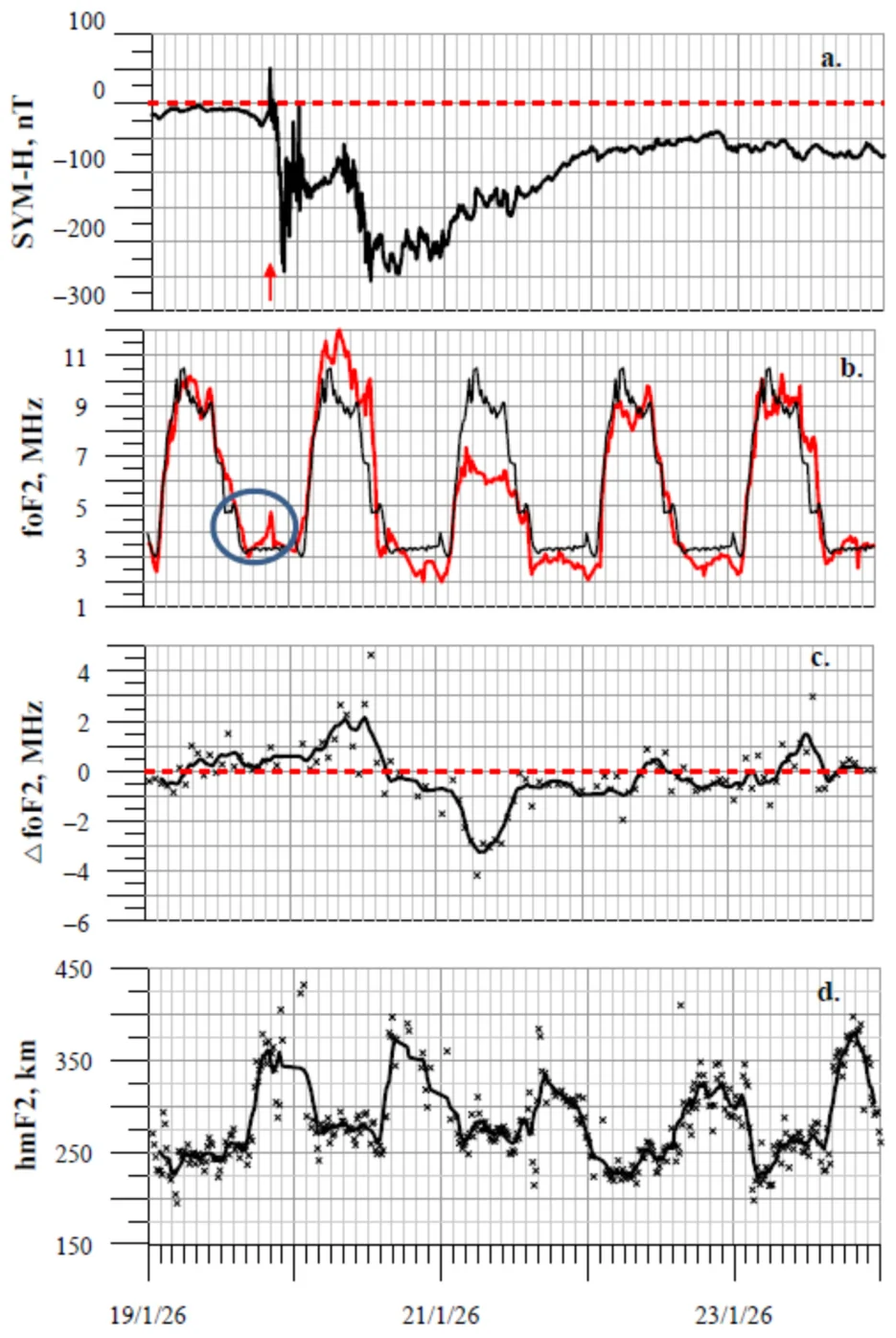
Variations in (**a**) SYM-H index; (**b**) critical frequency foF2 for the reference-day baseline (black solid line) and storm period (red solid line); (**c**) deviation ΔfoF2; and (**d**) peak height hmF2 during 19–23 January 2026. Symbol ↑ indicates the onset of the storm. The symbol “×” represents the variations observed from 19 to 23 January, while the solid curve represents the smoothed variations in ΔfoF2 and hmF2.

The impact of the strong geomagnetic storm of 19–21 January 2026, observed at the Almaty station on the ionosphere, is shown in Figure 2. The top panel (**a**) shows variations in the SYM-H index; the symbol “↑” indicates the onset of the storm. The second panel (**b**) shows diurnal UT variations in the critical frequency, foF2. The black solid line represents the variations on the reference day of 18 January, while the red solid line represents the variations observed from 19 to 23 January. Panel (**c**) shows the ionospheric storm effect, expressed as deviations of foF2 from the quiet-day level (ΔfoF2 = foF2_disturbed_ − foF2_quiet_). Panel (**d**) shows variations in hmF2. The symbol “×” represents the variations observed from 19 to 23 January, while the solid curve represents the smoothed variations in ΔfoF2 and hmF2.

**Figure 3:**
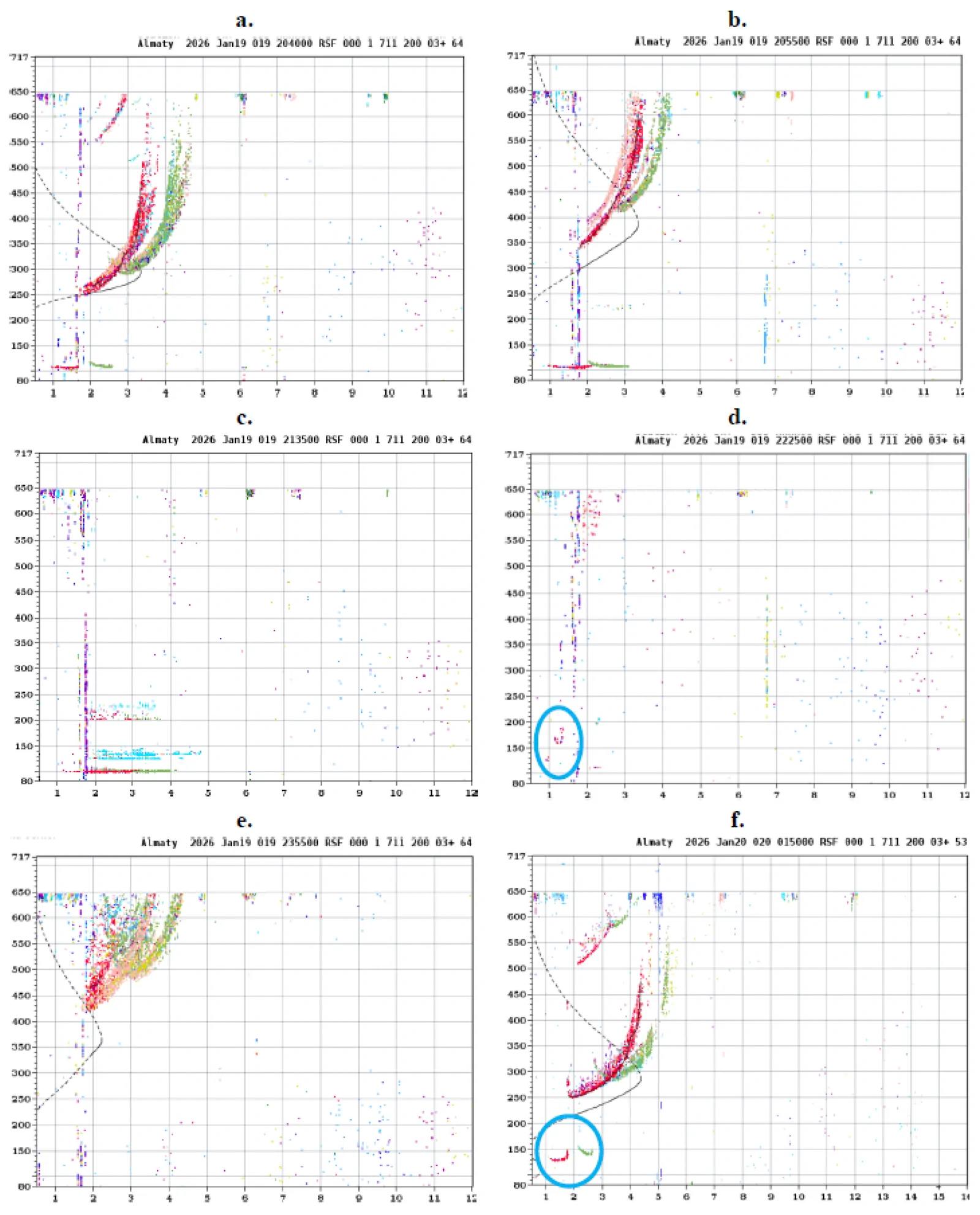
Sequence of DPS-4D ionograms over Almaty on 19–20 January 2026 showing spread-F (**a**,**b**,**e**,**f**), sporadic-E (**a**–**c**,**f**), and anomalous layers (panels (**d**,**f**), circled). Red and blue dots correspond to the ordinary component of reflected radio waves, and green dots to the extraordinary component. The black line represents the f(h) profile, where h is the true height.

As demonstrated in Figure 2, despite the onset of the storm main phase (MP1) near local midnight (LT = UT + 5 h), foF2 did not immediately exhibit a classic negative storm phase during the initial 7 h of high geomagnetic activity. Instead, the ionograms revealed localized structural perturbations:

– *Increasing in foF2 during the SSC:* A clear increase in foF2 during the SSC is visible in Figure 2 (circled).
– *Es layer and range-spread F*: At 20:35 UT on 19 January, a sporadic E layer (Es) formed at h′E ≈ 100 km (foEs ≈ 1.8 MHz). Concurrently, the virtual base height of the F layer (h′F) rose rapidly from 242 km to 345 km (Figure 3a,b) and continued to increase.
– *Blanketing Es and complete absence of reflected signals:* By 21:15 UT, a blanketing Es layer obscured the upper ionospheric layers (Figure 3c). Between 22:10 UT and 23:20 UT, there was a complete absence of reflected signals, interrupted only by weak, short-duration echoes near 150–160 km (Figure 3d).
– *Descending Diffuse Layers:* At 23:55 UT, high-altitude diffuse reflections appeared at 550–650 km (Figure 3e). These reflections descended and eventually reformed into a normal F-layer trace by 01:50 UT on 20 January (Figure 3f).

**Figure 4:**
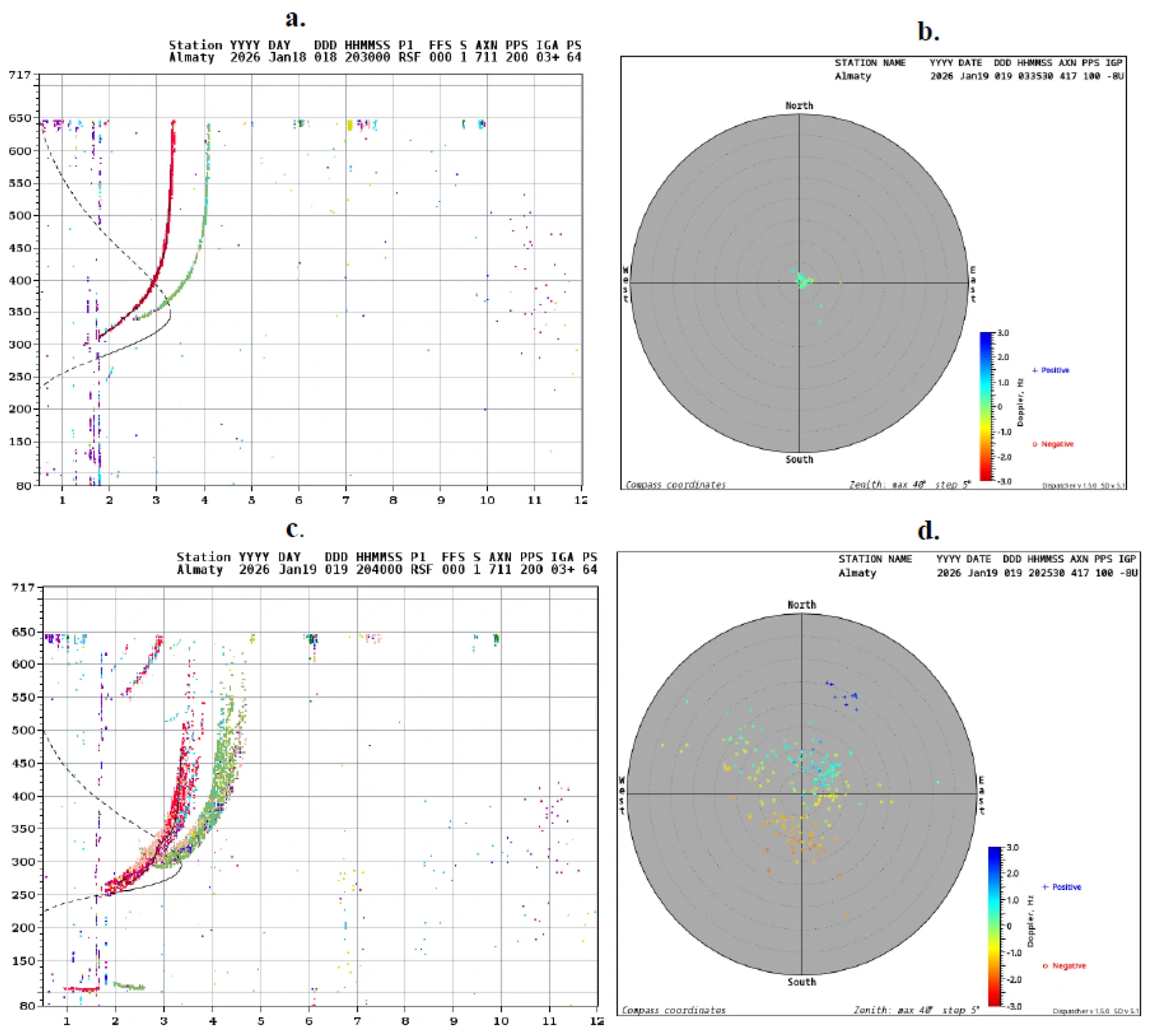
(**a**,**c**) DPS-4D ionograms and (**b**,**d**) polar “sky maps” for reference (18 January) vs. storm (19/20 January) night. The shape and color on the “sky maps” indicate the Doppler velocity (positive Doppler velocity is indicated by the “+” symbol and blue shades, and negative Doppler velocity by the “o” symbol and red shades).

Figure 4 shows examples of the DPS-4D ionograms (left panels) and “sky maps” (right panels) obtained at the Almaty station for reference (a, b) and disturbed nights (c, d) on 18/19 and 19/20 January 2026. We see that ionograms and sky maps contrast relatively quiet geomagnetic conditions (no Fsp, narrow zenith reflection cone) with storm conditions (Fsp, broad angular reflection cone with high Doppler spread, indicating strong electron density irregularities).

Then, following sunrise on 20 January (∼03:00 UT/08:00 LT), the ionosphere developed a biphasic response:

– *Positive Phase:* A strong positive storm phase persisted for ∼10 h (03:00–13:00 UT on 20 January). Peak foF2 increased by +21% (∼2 MHz above reference-day baseline).
– *Negative Phase:* Beginning around 18:45 UT on 20 January (∼23:45 LT), a prolonged negative phase developed and persisted through 21 January. foF2 decreased by 32% (∼3 MHz below the reference-day level).

**Figure 5:**
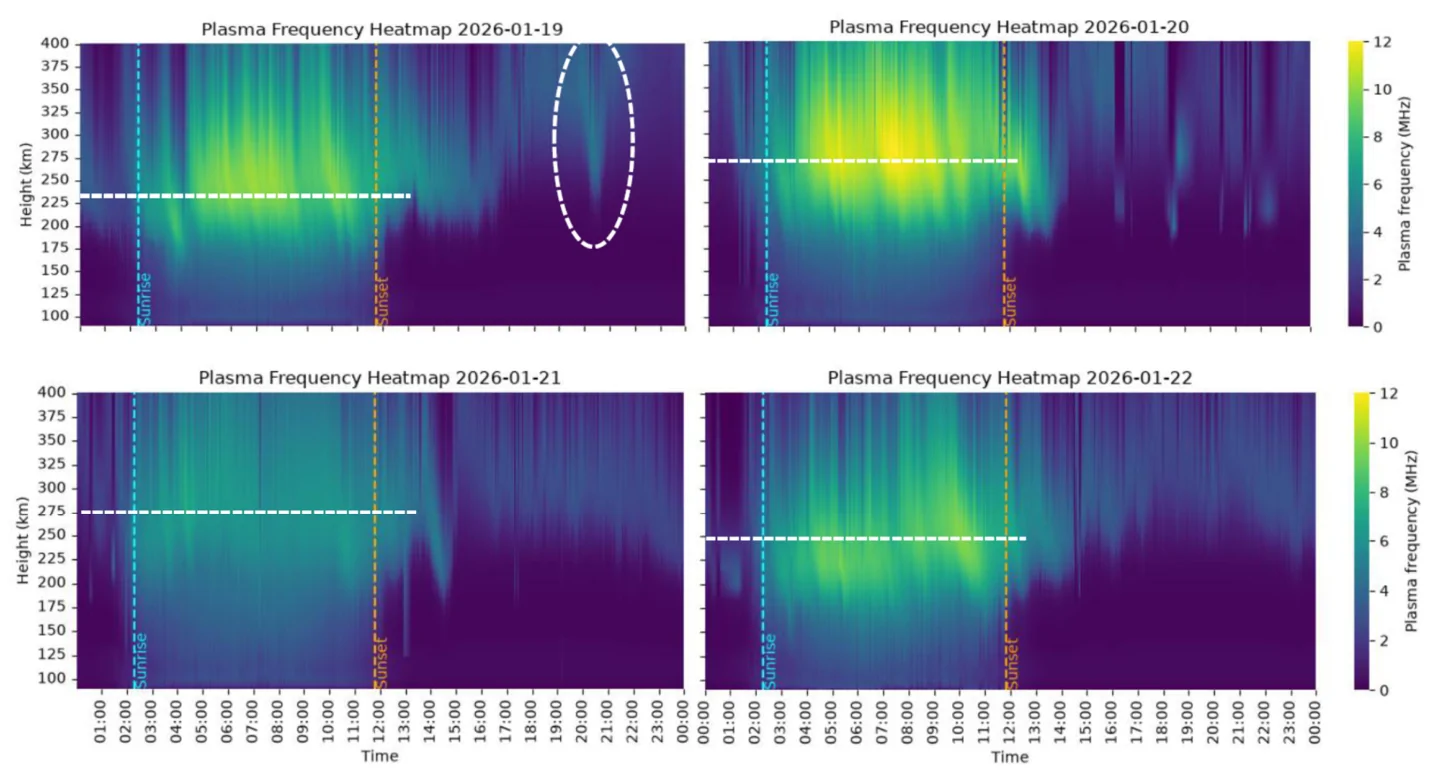
Changes in the electron density profile over the course of a day based on observations at the Almaty station on 19–22 January 2026. Electron density is represented as the equivalent plasma frequency (color bars). The vertical dashed lines indicate sunrise and sunset times. The horizontal dashed lines show the height of the maximum F-layer at 08:00 UT (13:00 LT). The circled section indicates an increase in foF2 during SSC.

**Figure 6:**
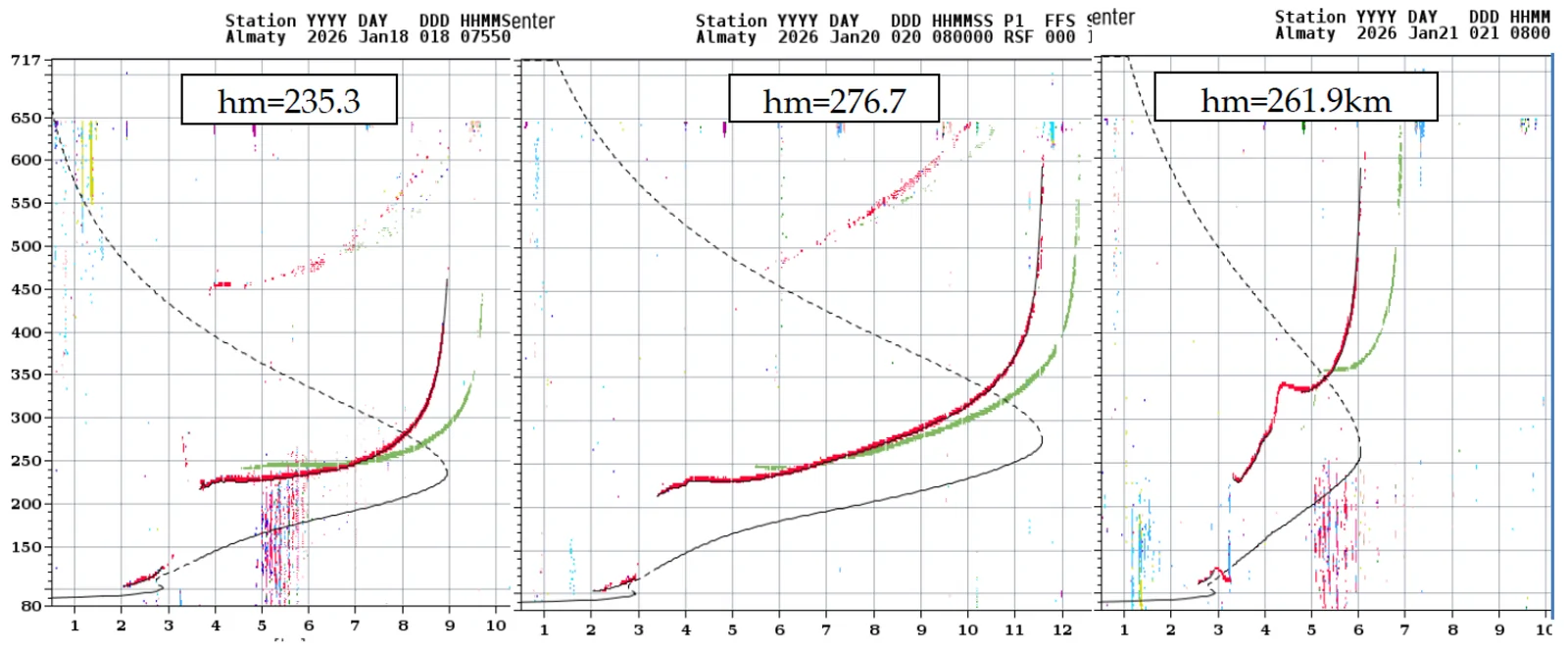
DPS_4D ionograms for noon-time on 18 January (at 07:55 UT), 20 January (at 08:00 UT), and 21 January (at 08:00 UT); hmF2—the height of the maximum F-layer.

During both phases, the peak layer height, hmF2, remained elevated (Figure 2d, Figure 5 and Figure 6). Figure 5 shows variations in the electron density profiles, N(h), while Figure 6 presents the corresponding noon ionograms.

**Figure 7:**
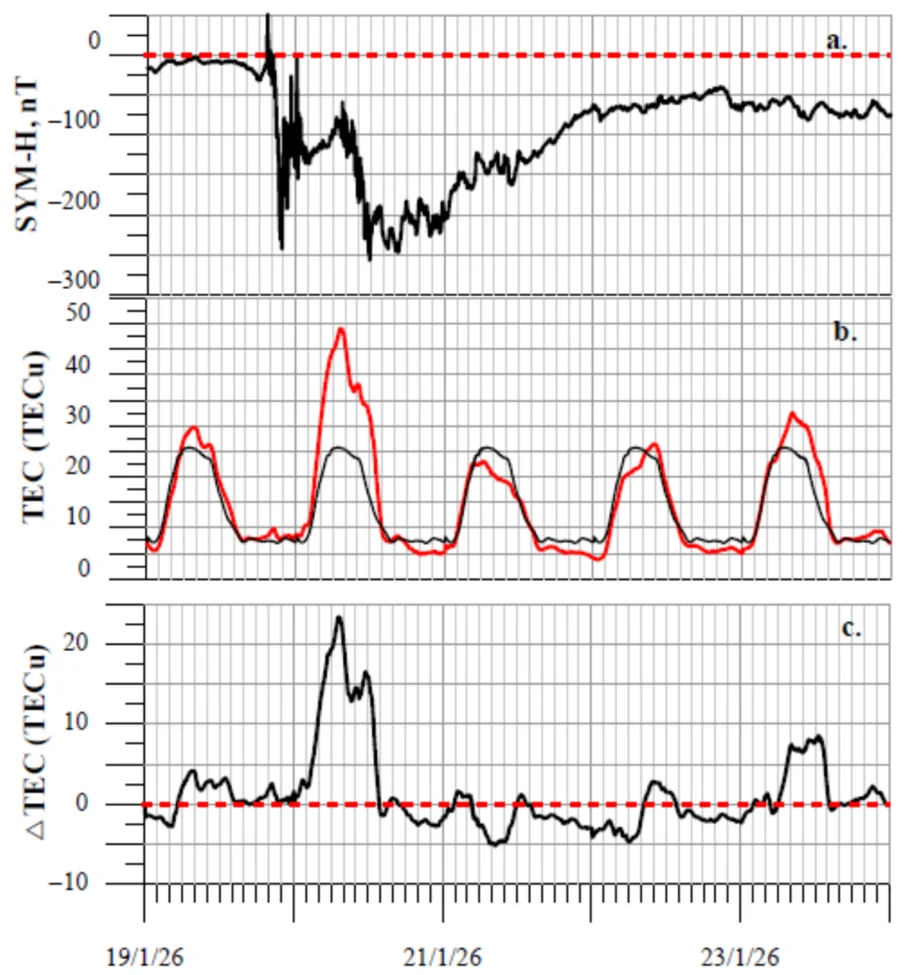
Variations in SYM-H (**a**), TEC (**b**), and ∆TEC (**c**). In the b-panel, the black solid curve represents the TEC variations on the reference day of 18 January; the red solid curve represents the TEC variations over the period from 19 to 23 January.

Figure 7 presents UT variations in the SYM-H index, total electron content (TEC), and the ionospheric storm effect, ΔTEC (the deviations of TEC from the reference-day level, defined as ΔTEC = TECdisturbed − TECref. day). The black solid curve in Figure 7b represents TEC variations on the reference day of 18 January, while the red solid curve represents the TEC variations during 19–23 January.

In general, Figure 2 and Figure 7 clearly show that, for this ionospheric storm event, the variations in the critical frequency and total electron content exhibit similar features. However, a direct comparison of their evolution reveals the following: while the total electron content increased by +92% at the peak of the positive disturbance (ΔTEC ≈ +25 TECU, where 1 TECU = 1016 el/m^2^), the peak of foF2 increased by +21% (∼2 MHz above the reference-day baseline). Conversely, while TEC decreased by ∼20% (TEC ≈ −5 TECU), foF2 decreased by ~32% (~3 MHz below reference level).

Without going into a detailed discussion of these phenomena, we show here that, despite the similarity in the behavior of foF2 and TEC during the ionospheric disturbance, their relative changes differ significantly. This difference may be due to the fact that the mechanisms governing the development of ionospheric disturbances in the bottomside (below hmF2) and topside (above hmF2) regions are different.

## 4. Discussion and Concluding Remarks

By analyzing two datasets, the F2 layer critical frequencies (foF2) and total electron content (TEC), obtained in Almaty (Kazakhstan, the Central Asian region) using data from the Almaty ground-based ionospheric station [43.25° N, 76.92° E] and the above-mentioned Chum GPS station [43.9° N, 74.75° E] (see Section 2 and Section 3), the ionospheric response to the strong geomagnetic storm of 19–21 January 2026 (SYM-H = −252 nT) was investigated. Before discussing the results of these observations, we briefly review the known mechanisms responsible for the formation of ionospheric disturbances during geomagnetic field disturbances.

The basic mechanisms of the formation of ionospheric storms that occur during geomagnetic disturbances are well known and have been described in detail in numerous studies [22,23,24,25,26,27,28,29]. It is generally accepted that the large amount of energy released into the thermosphere at high latitudes during geomagnetic disturbances leads to heating of the lower part of the thermosphere in the auroral zone, to an increase in the neutral gas temperature T, and to changes in the neutral composition (a decrease in the [O]/[N_2_] ratio). Both of these factors are the main cause of a decrease in the electron concentration Ne (the negative phase of an ionospheric storm) in the high-latitude ionosphere. Heating, in turn, causes its own circulation, which tends to move air equatorward to lower latitudes. However, this circulation can coincide in direction with or be opposite to the background circulation, which is determined by the time of day and season. As a result, the interference between storm-induced and normal circulation determines the spatial distribution of the electron concentration in different seasons, with Ne variations manifesting over a wide range of altitudes [30,31].

The ionospheric response to a geomagnetic storm can vary depending on the observation location coordinates, season, and time of day. Thus, for the summer season and mid-latitudes, during a geomagnetic storm, the most likely phase is the negative phase of the ionospheric storm, both daytime and nighttime. This is due to the nature of the background circulation system in the summer thermosphere, which is directed toward the equator for most of the day, coinciding with the storm-induced circulation [1]. For the winter ionosphere, this pattern of thermospheric circulation is different; it differs between daytime and nighttime conditions. During the daytime, the background and storm-induced circulations are opposite, and the negative phase of the storm is limited to high latitudes. However, at night, both circulations coincide in direction (both are directed towards the equator), which leads to the appearance of a negative phase of the ionospheric storm in the middle latitudes at night in winter.

**Figure 8:**
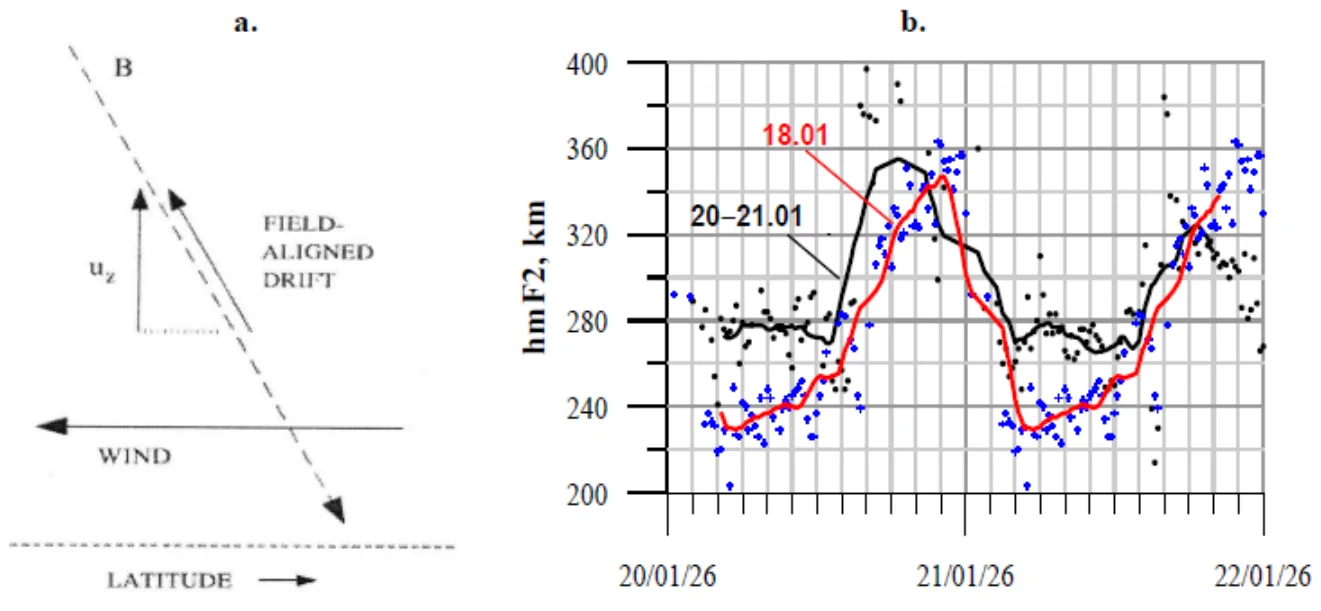
(**a**) Uplifting of the ionospheric F layer by an equatorward-directed wind, taken from [28]; (**b**) hmF2 variations during reference (18 January 2026) and disturbed conditions (20–21 January 2026).

Several mechanisms are considered as possible drivers of the positive disturbance. However, for midlatitudes, the most probable mechanism is assumed to be the influence of storm-induced equatorial thermospheric circulation, which lifts the F2 layer plasma along the magnetic field lines [1,32,33] and leads to the ascent of the F2 layer, as shown in Figure 8a. The situation illustrated applies to the northern hemisphere at a magnetic latitude of 45°; B is the Earth’s magnetic field, and u_z_ is the upward-directed component of the drift velocity. The ascent of the F2 layer leads to an increase in the ionization density as a result of a decrease in its losses at higher altitudes; the behavior of the hmF2 presented in Figure 8b does not contradict the above-described scheme.

**Figure 9:**
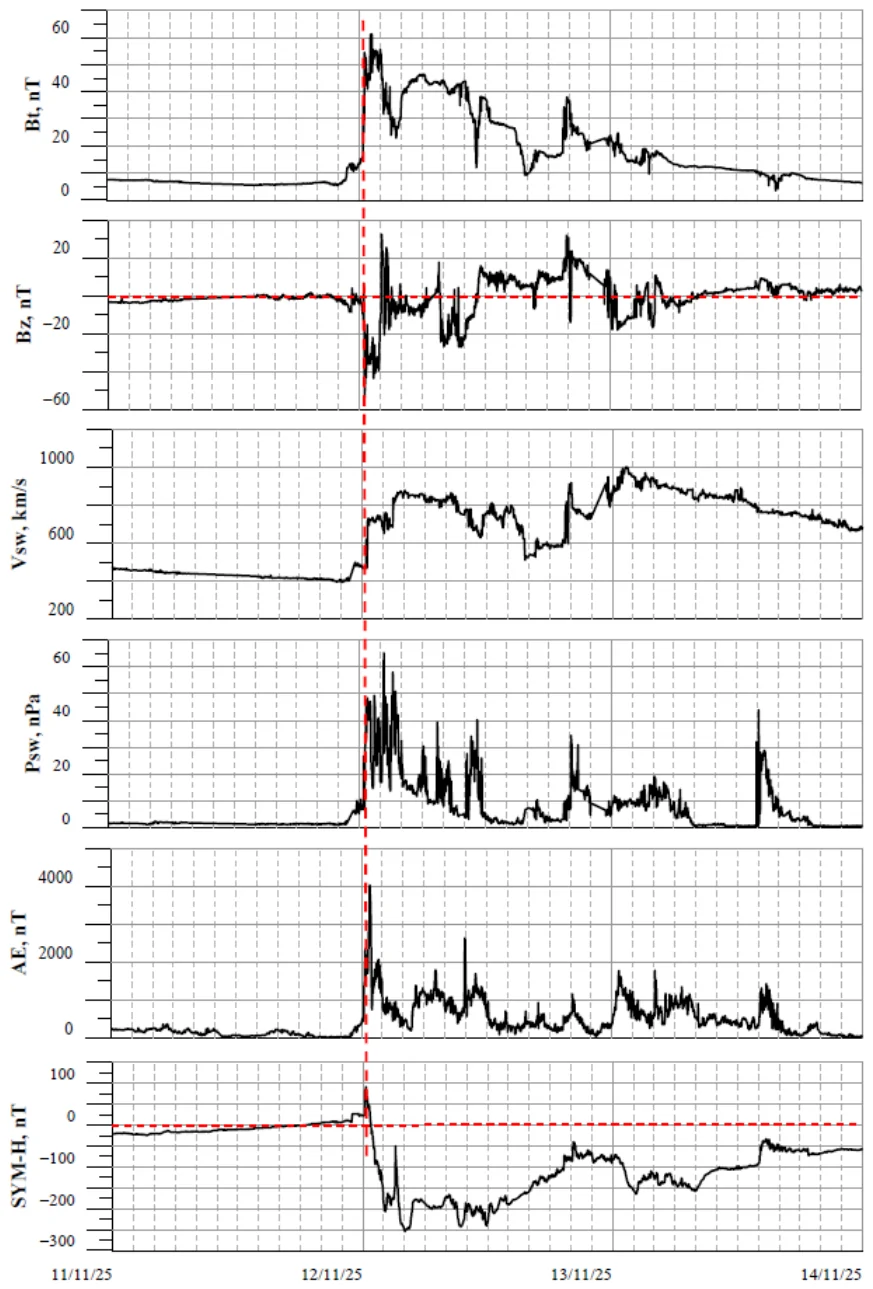
Time series (UT) of IMF components Bt and Bz, solar wind speed (Vsw), dynamic pressure (Psw), auroral electrojet index (AE), and SYM-H index for 10–15 November 2025. The Storm Sudden Commencement (SSC) is indicated by a vertical dashed line.

For further discussion of the obtained results, an attempt was made to analyze the ionospheric response to the geomagnetic storm event of 11–13 November 2025, observed over Almaty. This event had similar characteristics (sudden onset at night during the winter season, two-stage development, and storm class), allowing a comparison of its dynamics with those of the January 2026 event. A series of solar flares on 8–10 November 2025, including X1.8 (07:35 UTC, 9 November) and X1.2 (09:19 UTC, November 10) class flares, accompanied by successive coronal mass ejections (CMEs), formed a chain of CMEs that reached Earth, resulting in a severe storm (level G4) on 12 November. The helio-geophysical parameters recorded on 11–15 November 2025, when the geomagnetic storm occurred, are presented in Figure 9.

The Storm Sudden Commencement (SSC) occurred at 00:16 UT on 12 November, which is indicated by a vertical dashed line, and manifested itself as a sudden impulse SYM-H (Sudden Impulse (SI)) with an amplitude ΔSYM-H = +92 nT. Figure 9 shows the IMF component (Bt, Bz), solar wind plasma speed (Vsw), plasma density (Psw), and the AE and SYM-H indices. At the storm commencement time, the solar wind velocity increased to ~740 km/s, accompanied by high dynamic pressure Psw and an increase in IMF-Bt. The IMF-Bz component demonstrated rapid, large-amplitude oscillations and a southerly direction with a peak value of −54.49 nT at 00:28 UT on 12 November. High AE values indicated a sustained energy injection into the high-latitude magnetosphere.

Approximately at this time, the main phase of the storm began, with the SYM-H index reaching its minimum at 03:59 UT over an approximately 4 h interval. The minimum of −254 nT indicated a severe geomagnetic storm. The impact of the entire CME chain resulted in a fairly long (about 9 h) period of low SYM-H values, characterized by significant fluctuations that correlated in duration with increased Psw and AE activity, while IMF-Bz during this period remained predominantly southerly (−60 ≤ Bz ≤ −20 nT).

After ~12:00 UT on 12 November, SYM-H values increased, and the recovery phase of the geomagnetic field began. However, later that day, the third coronal solar mass ejection generated by the X5 flare (10:04 UT, 11 November) reached the Earth’s magnetic field at approximately 19:17 UTC. This caused an additional impact on the development of the geomagnetic storm during its recovery phase, leading to an increase in geomagnetic activity to a G3 level (−165 nT) at ~02:12 UT on 13 November. This increase is clearly associated with a significant level of negative IMF-Bz values (about −15 nT) sustained for at least 4 h.

**Figure 10:**
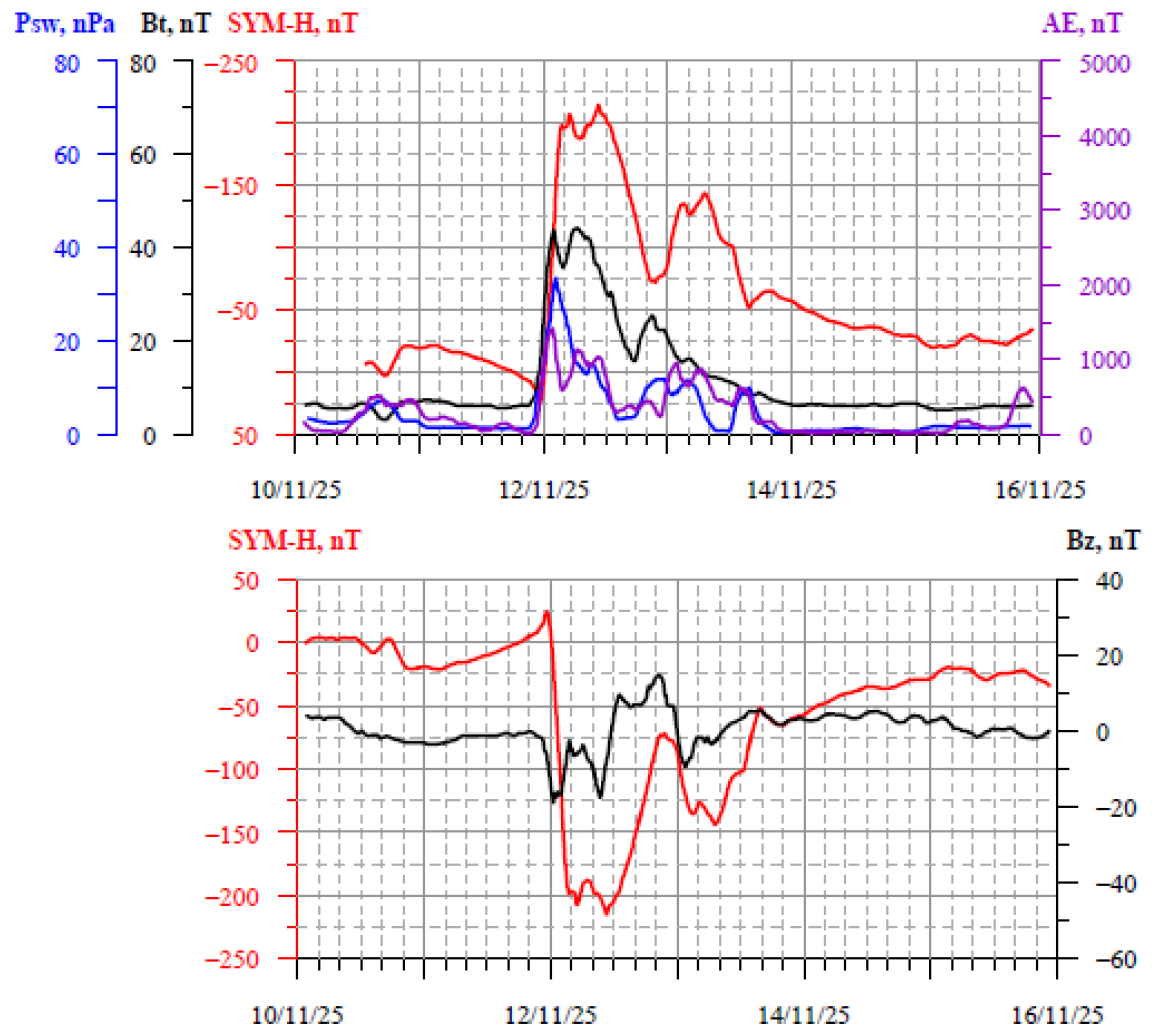
Time series (UT) of smoothed IMF components Bt and Bz, dynamic pressure (Psw), auroral electrojet index (AE), and SYM-H index for 10–15 November 2025.

In general, the figure shows the temporal evolution of the geomagnetic and solar-wind parameters during the 12–13 November geomagnetic storm. In this particular event, the durations of the individual stages of the disturbed period and the overall storm interval appear to be temporally associated with periods of enhanced variations in Bt, Bz, and Psw. To facilitate comparison of the temporal behavior of the parameters and reduce short-term fluctuations in the minute-resolution data, the time series were smoothed using a moving average with a window of (W = 180) (3 h). Figure 10 shows a temporal offset between the extreme of SYM-H and those of the other parameters: the SYM-H minimum occurred approximately 1 h 40 min after the corresponding peak variations in Bt, Psw, Bz, and AE. These relationships are presented here as descriptive temporal features of the 12–13 November event and should not be interpreted as evidence of a statistically established correlation or as a general quantitative relationship between the parameters.

Ionospheric behavior during the storm event is compared with a reference day (10 November, Ap = 14, Kp = 2.7) to identify possible storm impacts. We examined the evolution of foF2 and TEC characteristics to quantify the positive and negative effects of the storm and compare them with the effects observed during the storm of 19–21 January 2026. Notably, the Digisonde operated in a 15 min test mode in November; thus, we use 15 min for foF2 and TEC data.

**Figure 11:**
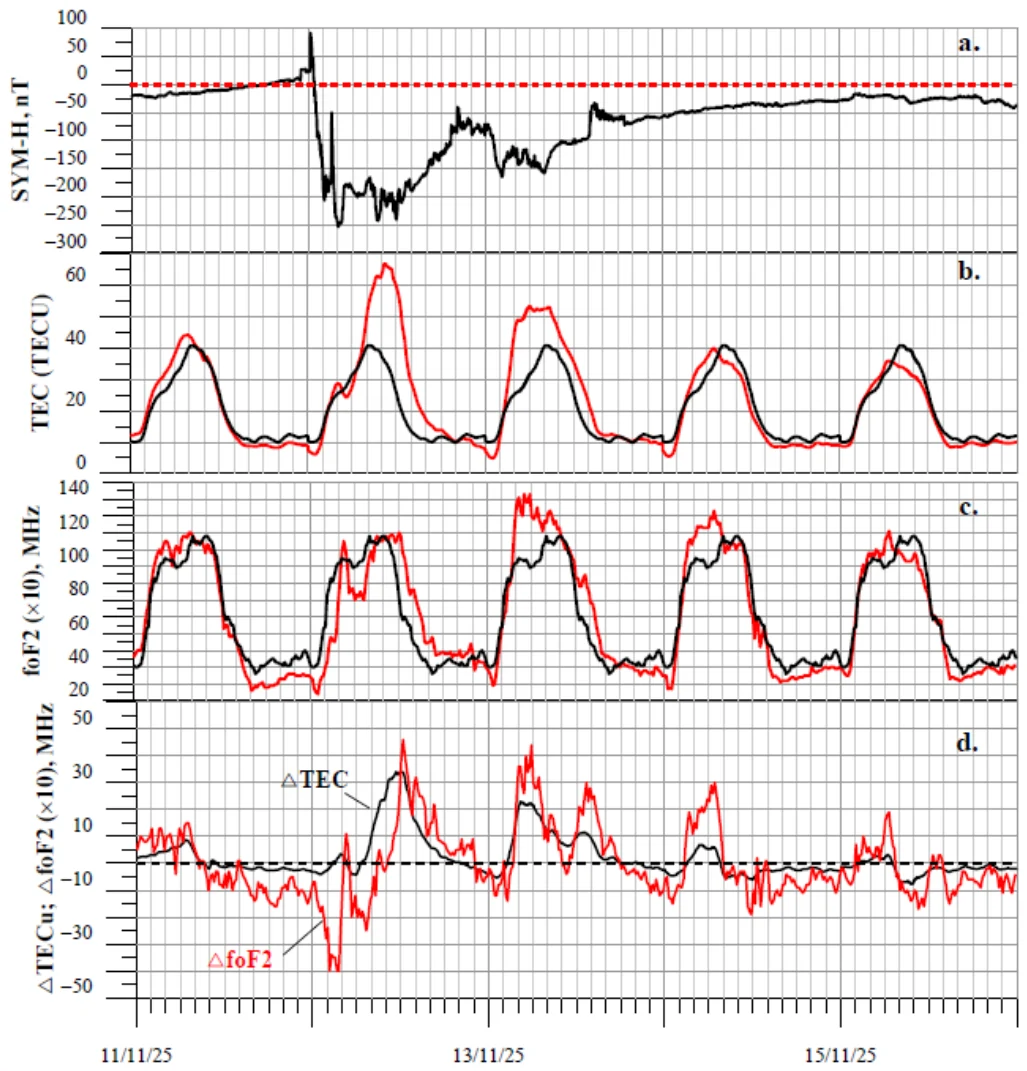
Variations in (**a**) SYM-H index; (**b**) total electron content TEC and (**c**) critical frequency foF2 for the reference-day baseline (black solid line) and storm period (red solid line); (**d**) deviation ΔfoF2 and TEC during 11–15 November 2025.

Figure 11 shows the diurnal UT variations in SYM-H (a), total electron content TEC (b), critical frequency foF2 (c), and their variabilities ∆TEC and ∆foF2 (d), obtained from the Chum and Almaty stations for the period 11–15 November 2025. The black solid lines in Figure 11b,c represent the reference-day variations, while the red solid lines represent the variations in TEC and foF2 for 11–15 November. As a reference, the SYM-H index is plotted in the top panel, showing the storm commencement, stages, and recovery phases.

As shown in Figure 11, the ionosphere showed the following structural changes in response to the geomagnetic storm:

– A marked increase in TEC values (a positive phase of ionospheric disturbance), which began at ~07:00 UT (~12:00 LT) on 12 November with a ~7 h delay relative to the onset of the geomagnetic storm. The duration of the increased TEC values was about 8 h (08:00–16:00 UT); the maximum increase in TEC was about 143% (∆TEC ≈ +33 TECU) relative to the reference level (where 1 TECU = 10^16^ el/m^2^).
– A two-phase (−/+) disturbance in foF2 on 12 November:
  - A decrease in foF2 values (a classic negative phase of disturbance) that began synchronously with the onset of a geomagnetic storm. The duration of the decreased foF2 values was ~10 h (00:16–10:00 UT), and the maximum decrease in foF2 values reached ~4 MHz.
  - An increase in foF2 values at ~10:00 UT (change from the negative phase to the positive disturbance phase) with a 3 h delay relative to the onset of the positive disturbance in TEC. The maximum change in critical frequencies was ~4.0 MHz (ΔfoF2 ≈ 53%, or ΔNmF2 ≈ 135%). By the end of the day on 12 November, the critical frequency values had reached the reference-day level.
– A synchronous positive disturbance in TEC and foF2 variations occurred on 13 November. The maximum changes in TEC and foF2 were +20 TECU (∆TEC ≈ 80%) and 4 MHz (∆foF2 ≈ 40%, or ∆NmF2 ≈ 96%), respectively. The disturbance duration on November 13 was ~12 h (04:00 UT–16:00 UT).

A comparative analysis of ionospheric responses to two severe geomagnetic storms in January 2026 and November 2025 showed that:

(1) The positive storm effects tend to start between 08:00 LT and 15:00 LT (positive storms in TEC and foF2 during the November 2025 event and a positive storm in TEC on 20 January 2026), while negative effects were found to begin around midnight (e.g., at 23:45 LT on 20 January 2026) or after midnight (e.g., at 05:16 LT on 12 November 2025). The increase in ionization density was accompanied by a significant increase in the F2-layer altitude. These ionospheric responses to the observed geomagnetic storms generally corresponded to the above-described scenario of the development of ionospheric storms during the winter season. Nevertheless, certain discrepancies also emerge:
  ▪ No significant changes were observed in either the foF2 critical frequency or the total electron content (TEC) during the main phase MP1 of the January geomagnetic storm. Given that the storm’s sudden commencement (SSC) occurred near local midnight in winter (19:24 UT on January 19/00:24 LT on 20 January), classical storm theory [1,30] predicts an immediate negative phase caused by the equatorward transport of thermospheric heated-composition zones from high latitudes (i.e., regions with a reduced [O]/[N2] ratio). However, foF2 and TEC remained near reference-day levels for the first 7 h, despite the occurrence of spread-F, layer uplift, and other features. This behavior, when compared with the prolonged negative ionospheric storm (sustained for ~10 h) during MP1 of the November geomagnetic storm, indicates that MP1 of the January geomagnetic storm (duration ∼2.3 h) was insufficient to allow the development of an ionospheric storm and the advection of low-[O]/[N2] air to mid-latitudes before sunrise.
  ▪ A prolonged negative phase in foF2 and TEC variations finally occurred at 18:45 UT/23:45 LT on 20 January 2026 and lasted for more than 24 h, spanning both nighttime and daytime. This may indicate that storm-induced changes in circulation and thermospheric composition persisted in the ionosphere for a long time due to the prolonged influence of CME effects and sustained southward Bz activity, which remained predominantly southerly, reaching values of about −10 nT or below, with periodic peaks around −30 nT.
(2) The observed delays in the positive storm effects were ~2 h and 7–10 h relative to the SSC, whereas the negative effect of the ionospheric storm can begin simultaneously with the SSC or with a delay of up to 9 h. These results suggest a potential dependence of the time delay of the ionospheric storm effect on the local time of the geomagnetic storm commencement; however, analysis of a larger number of events is necessary to firmly establish such a relationship.
(3) The behavior of the critical frequency and total electron content during geomagnetic storms exhibits both common features (i.e., a general response to a geomagnetic storm) and notable differences. In particular, differences can be observed in the relative changes (with respect to the reference-day level) in foF2 and TEC. Although these changes may have the same sign, as in the January 2026 event, they can also exhibit complex dynamics, as observed during the November 2025 event, indicating an ambiguous relationship between them. Thus, during the January 2026 event, the positive phase exhibited a ~92% increase in TEC compared with a ~21% increase in foF2 (~46% in NmF2), whereas the negative phase showed a ~20% decrease in TEC alongside a ~32% decrease in foF2 (~53% in NmF2). During the November 2025 event, the relative changes in TEC and foF2 (NmF2) were approximately +143% and +53% (135%), respectively, on 12 November, and +80% and +40% (96%), respectively, on 13 November.
(4) An interesting effect we observed is a difference between the storm-time disturbances in foF2 and TEC during the main phase MP1 of the November 2025 storm, namely, the development of a pronounced positive disturbance in TEC and a two-phase (−/+) disturbance in foF2 on 12 November 2025. This effect may indicate a competition between the mechanisms responsible for positive and negative ionospheric effects, greater variability in the bottomside ionosphere (below hmF2), or that other processes became dominant.
(5) The next interesting effect we observed is some night-time anomalous, short-duration wave-like echoes at the E or F1 region heights at our midlatitude station, which could probably be related to the ionization production by precipitated neutralized ring current particles, as explained by the Bauske et al. [34] simulation.

Recently, Pulinets et al. [15] examined global ionospheric dynamics during the 12–14 November 2025 geomagnetic storm using the multiparameter measurements from the LAERT sounders and data from Roshydrometʹs network of ground-based ionosondes located across the European longitude sector (Rostov-on-Don, Moscow, and Kaliningrad), as well as in the Far Eastern region (Khabarovsk, Magadan, and Petropavlovsk-Kamchatsky). The authors reported an extended negative phase of the storm over the Rostov-on-Don station that began synchronously with the geomagnetic disturbance and lasted for about 24 h (from 03:00 UT (06:00 LT) on 12 November to 03:00 UT (06:00 LT) on 13 November 2025 (Figure 3 in [15], upper panel)). This ionospheric behavior was also typical of other stations within the same longitudinal sector, specifically Kaliningrad and Moscow. On 13 November 2025, a minor increase in the critical frequency was observed later in the day. For Khabarovsk and other locations within the same longitudinal sector, however, the character of the ionospheric variations differs significantly. A 20% increase in the ionospheric critical frequency was observed on 12 November, marking the positive phase of the storm, followed by a 30% decrease in the critical frequency that persisted for nearly another full day (Figure 3 in [15], bottom panel). Our results demonstrate an “intermediate pattern” over Almaty: on 12 November, a negative phase in foF2 began during the nighttime, synchronously with the SSC (as in Rostov-on-Don), followed by an increase in foF2 during the daytime on the same day (as in Khabarovsk). On 13 November, similar variations in foF2 were observed in Almaty and Rostov-on-Don, but opposite variations occurred in Almaty and Khabarovsk.

***Concluding remarks.*** This study provides the first detailed investigation of mid-latitude ionospheric storm dynamics over Almaty, Kazakhstan, based on combined observations from a DPS-4D Digisonde and GPS-VTEC measurements during the severe geomagnetic storm of 19–21 January 2026. The ionospheric dynamics during the geomagnetic storm of 12–13 November 2025 were additionally analyzed to compare the obtained results in order to obtain an overall picture of the ionospheric effects within the latitude–longitude region under consideration.

Our analysis shows that the formation and evolution of ionospheric storms are highly complex processes. The behavior of the critical frequency and total electron content during severe geomagnetic storms exhibits both common features (i.e., a general response to geomagnetic storms) and notable differences, including differences in their storm-time responses and in the observed relative changes in foF2 and TEC with respect to reference-day levels. Although these changes may have the same sign, they can also exhibit complex and dissimilar dynamics. This demonstrates that relative variations in foF2 and TEC are sensitive indicators of ionospheric conditions during geomagnetic storms. Investigating these variations is important not only for the correct interpretation of observational data but also for understanding the complex physical processes occurring in the ionosphere.

Along with the dependence of the ionospheric response on local time, its behavior during geomagnetic disturbances can be determined by possible longitudinal variations in temperature, dynamics, and the ΣO/N_2_ ratio at mid-latitudes. Consequently, changes in foF2 during geomagnetic storms can be local in nature. The location of the Almaty station—the only station in Central Asia—provides access to critical ionospheric observation data for the region, which are essential for studying large-scale space weather processes.

Of course, the analysis of only two storms does not provide a sufficiently large dataset, and further research is required to obtain statistically significant results. This work is planned to be carried out using a larger body of experimental data.

## Data Availability

The TEC data used in this study were obtained from the publicly accessible IONOLAB service (http://www.ionolab.org, (accessed on 14 September 2026)). Solar wind and geomagnetic indices were obtained from the NASA/GSFC OMNIWeb database (https://omniweb.gsfc.nasa.gov/form/omni_min.html (accessed on 14 September 2026)). This study utilized data from the ionosonde at the Institute of the Ionosphere (Almaty, Kazakhstan); all ionograms from January 2026 used in this work are available in the GIRO repository (https://giro.uml.edu; accessed 3 January 2025); for further information regarding the ionospheric data used in this study, please contact G. Gordiyenko (ggordiyenko@mail.ru).
